# Myeloid differentiation 2 deficiency attenuates AngII-induced arterial vascular oxidative stress, inflammation, and remodeling

**DOI:** 10.18632/aging.202402

**Published:** 2021-01-20

**Authors:** Shushi Huang, Shengban You, Jinfu Qian, Chengyi Dai, Siyuan Shen, Jun Wang, Weijian Huang, Guang Liang, Gaojun Wu

**Affiliations:** 1 Department of Cardiology, The First Affiliated Hospital, Wenzhou Medical University, Wenzhou, Zhejiang, China; 2 Chemical Biology Research Center, School of Pharmaceutical Sciences, Wenzhou Medical University, Wenzhou, Zhejiang, China; 3 Department of Cardiology, Affiliated Dingli Institute and Wenzhou Central Hospital, Wenzhou Medical University, Wenzhou, Zhejiang, China

**Keywords:** vascular remodeling, MD2, inflammation, oxidative stress, VSMCs

## Abstract

Vascular remodeling is a pertinent target for cardiovascular therapy. Vascular smooth muscle cell (VSMC) dysfunction plays a key role in vascular remodeling. Myeloid differentiation 2 (MD2), a cofactor of toll-like receptor 4 (TLR4), is involved in atherosclerotic progress and cardiac remodeling via activation of chronic inflammation. In this study, we explored the role of MD2 in vascular remodeling using an Ang II-induced mouse model and cultured human aortic VSMCs. MD2 deficiency suppressed Ang II-induced vascular fibrosis and phenotypic switching of VSMCs without affecting blood pressure in mice. Mechanistically, MD2 deficiency prevented Ang II-induced expression of inflammatory cytokines and oxidative stress in mice and cultured VSMCs. Furthermore, MD2 deficiency reversed Ang II-activated MAPK signaling and Ang II-downregulated SIRT1 expression. Taken together, MD2 plays a significant role in Ang II-induced vascular oxidative stress, inflammation, and remodeling, indicating that MD2 is a potential therapeutic target for the treatment of vascular remodeling-related cardiovascular diseases.

## INTRODUCTION

Vascular remodeling is the hallmark of many cardiovascular diseases including hypertension and atherosclerosis. It may cause vessel narrowing, increase vascular resistance, augment wall stiffness, or–on the contrary–augment wall distensibility, leading to aneurysm development or atherosclerotic plaque progress and rupture [1]. Pathological vascular remodeling contributes to reduced vessel compliance that exacerbates cardiovascular diseases. Therefore, vascular remodeling is a pertinent target for cardiovascular therapy. The key mechanisms of vascular remodeling involve smooth muscle cell phenotypic switching, migration, apoptosis, and abnormal collagen turnover [2]. Vascular remodeling displays a close connection with the inflammation-related state and oxidation stress [3, 4]. Pro-inflammatory cytokines such as interleukin-6 (IL-6) and tumor necrosis factor-α (TNF-α) and increased ROS level could activate the nuclear factor-κB (NF-κB) pathway which further execrates proinflammatory factor expression and induces the expression of genes related vascular remodeling [3–5].

Vascular remodeling is the main pathological process in a series of hypertensive complications. Angiotensin II (Ang II) refers to the main effecting element inside the renin-angiotensin system (RAS), critically impacting the regulation of blood pressure, development of hypertension and vascular remodeling, as well as the induction of vascular inflammation and oxidation stress inducing processes [6–8]. The mentioned activities receive the mediation in resident vascular cells, covering endothelial cells and vascular smooth muscle cells (VSMCs). According to clinical studies, drugs suppressing the renin angiotensin-aldosterone system (RAAS), specifically the synthesis of Ang II (ACE inhibitors) or the binding of the ligand to its receptor (ARBs), are beneficial in reversing conductive vascular remodeling because of hypertension [9].

Myeloid differentiation 2 (MD2) was originally identified as a cofactor pertaining to toll-like receptor 4 (TLR4), facilitating lipopolysaccharide (LPS) to be recognized [10]. Recently, we found that MD2 promoted atherosclerosis via mediating TLR4 activating process under the induction by ox-LDL and inflammation-related cytokine expressing state in macrophages [11]. Ox-LDL could induce MD2/TLR4 complex formation and inflammatory responses via directly binding to MD2 protein. Interestingly, Ang II could also directly bind to MD2, inducing cardiac inflammation and remodeling byactivatingTLR4/NF-κB signaling channel [12]. Nevertheless, whether MD2 impacts vascular remodeling under the induction by Ang II is still unknown. This study aimed to examine the effect exerted by MD2 in vascular remodeling, ROS generation and inflammation-related responses in an Ang II-induced mouse model and cultured human aortic vascular smooth muscle cells (VSMCs).

## RESULTS

### Ang II elevated MD2 level of VSMCs in mice

**Figure 1:**
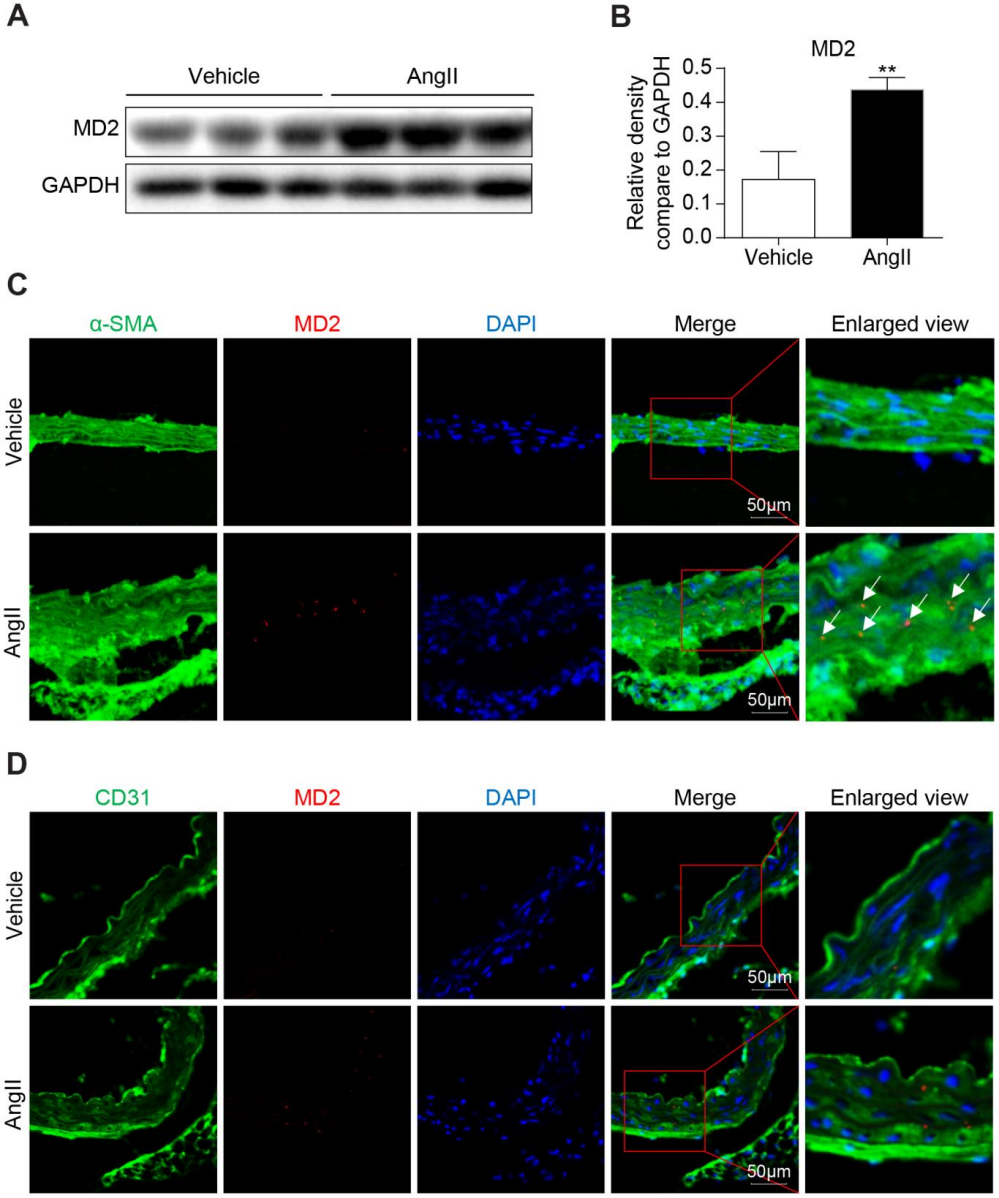
**MD2 is increased in aortas of Ang II induced mice.** (**A**) MD2 protein levels in aortas of mice induced by Ang II were detected by western blotting. Panel (**B**) showed densitometric quantification (n = 10; **P<0.01 compared to Vehicle). (**C**) Representative immunofluorescence staining of MD2 (red) and VSMCs marker α-SMA (green). Tissues were counterstained with DAPI (blue). Yellow arrows indicate co-location of MD2 and α-SMA stanning (scale bar = 50 μm). (**D**) Representative immunofluorescence staining of MD2 (red) and endothelial cells marker CD31 (green). Tissues were counterstained with DAPI (blue). Yellow arrows indicate co-location of MD2 and CD31 stanning (scale bar = 50 μm).

The pathological progress of vascular remodeling was induced by 2-week AngII subcutaneous micropump in mice. This model for vascular remodeling has been widely used in previous researches [13, 14]. We also confirmed the AngII level in serum of Ang II-challenged mice was significantly increased (Supplementary Figure 1). In an Ang II-induced vascular remodeling mouse model, the aortas were excised for Western blot and immunofluorescent staining of MD2. MD2 protein level in the aortas was remarkably increased in Ang II- administrated mice in comparison with control mice (Figure 1A, 1B). The increased MD2 expression was colocalized with a VSMC marker, α-SMA, but not endothelial cells (ECs) marker, CD31, (Figure 1C, 1D). Also, the MD2 positive area was significantly increased in Ang II-administrated mouse aortas (Supplementary Figure 2). Similar to our previous study of co-localized MD2 immunoreactivity with cardiomyocyte in heart tissues [15], MD2 immunoreactivity dots in aortas tissue sections were dispersed and distributed in the cell surface. The immunofluorescent staining of MD2 in aortas was consistent with the biological distribution of MD2, which, as an assistant protein of TLR4, functions in the outside of cell membrane (Figure 1C, 1D).

### MD2 deficiency alleviated Ang II-induced vascular remodeling *in vivo*

**Figure 2:**
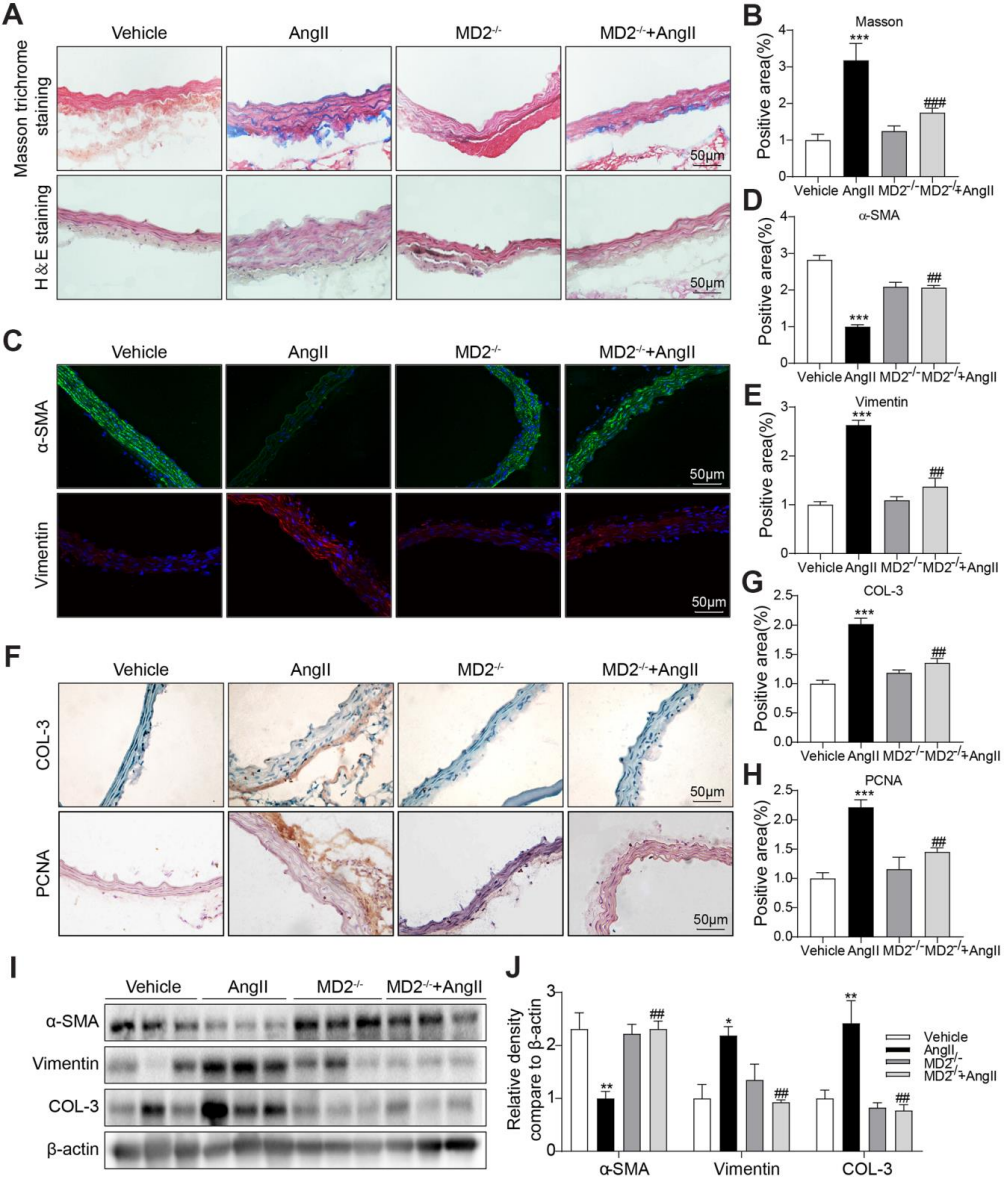
**MD2 deficiency alleviated Ang II induced vascular remodeling *in vivo*.** (**A**) Aortic thickening in the mice and aortic tunica media fibrosis was examined by Masson trichrome staining and H&E staining (scale bar = 50 μm). (**B**) Quantification for staining results in 2A (n = 10; ***p<0.001 compared to Vehicle; ###p<0.001 compared to Ang II). (**C**) Representative immunofluorescence staining images for α-SMA (green) and Vimentin (red) in aortas. Tissues were counterstained with DAPI (blue) (scale bar = 50 μm). (**D**, **E**) Quantification for staining results in 2C (n = 10; ***p<0.001 compared to Vehicle; ##p<0.01 compared to Ang II). (**F**) Representative images of COL-3 and PCNA staining of aortas (scale bar = 500 μm; DAB chromogen staining (brown). (**G**, **H**) Quantification for staining results in 2F (n = 10; ***p<0.001 compared to Vehicle; ##p<0.01 compared to Ang II). (**I**, **J**) Expressions of α-SMA, Vimentin and COL-3 in the whole aorta (n = 10; *p<0.05, **p<0.01 compared to Vehicle; ##p<0.01 compared to Ang II).

To identify the role of increased MD2 in vascular remodeling, MD2-KO mice and their WT littermates were subjected to the Ang II-induced vascular remodeling model. Ang II infusion equally increased systolic blood pressure in the WT and MD2 knockout mice (Supplementary Figure 3), indicating that MD2 deficiency did not affect the level of blood pressure. Similar result was observed in our previous study [12]. Furthermore, blockade of TLR4 has been found to fail to change blood pressure in both Ang II-injected TLR4^-/-^ mice [16] and adult spontaneously hypertensive rats [17]. These results indicate that MD2/TLR4 innate immune signaling is not involved in AngII-induced hypertension. In these hypertensive mice, Masson trichrome staining in the aortic tissue showed that Ang II infusing process noticeably improved collagen deposition in the vascular wall of WT mice, while the vascular collagen deposition was not observed in MD2-KO mice (Figure 2A, 2B). According to H&E staining, medial wall thickness was elevated after Ang II infusion in the WT mice, instead of MD2-KO mice (Figure 2A). Further immune staining against α-SMA (a contractile phenotype marking element pertaining to VSMCs) and Vimentin (a synthetic phenotype marker of VSMCs) showed that α-SMA was significantly decreased and Vimentin was increased after Ang II infusion in the WT mice but not MD2-KO mice (Figure 2C–2E). Additionally, proliferating cell nuclear antigen (PCNA) and the pro-fibrotic protein collagen III (COL3) were also reduced in MD2-KO mice compared to the WT mice with Ang II infusion (Figure 2F–2H). The changes in α-SMA, Vimentin and COL3 were further confirmed Western blot (Figure 2I, 2J). These results indicated that MD2 deficiency reduced the VSMC phenotypic switching and collagen depositing process in the aortas of Ang II infusion mice via non-hypotensive mechanisms.

### MD2 deficiency alleviated Ang II-induced inflammation and oxidative stress in the aortas

**Figure 3:**
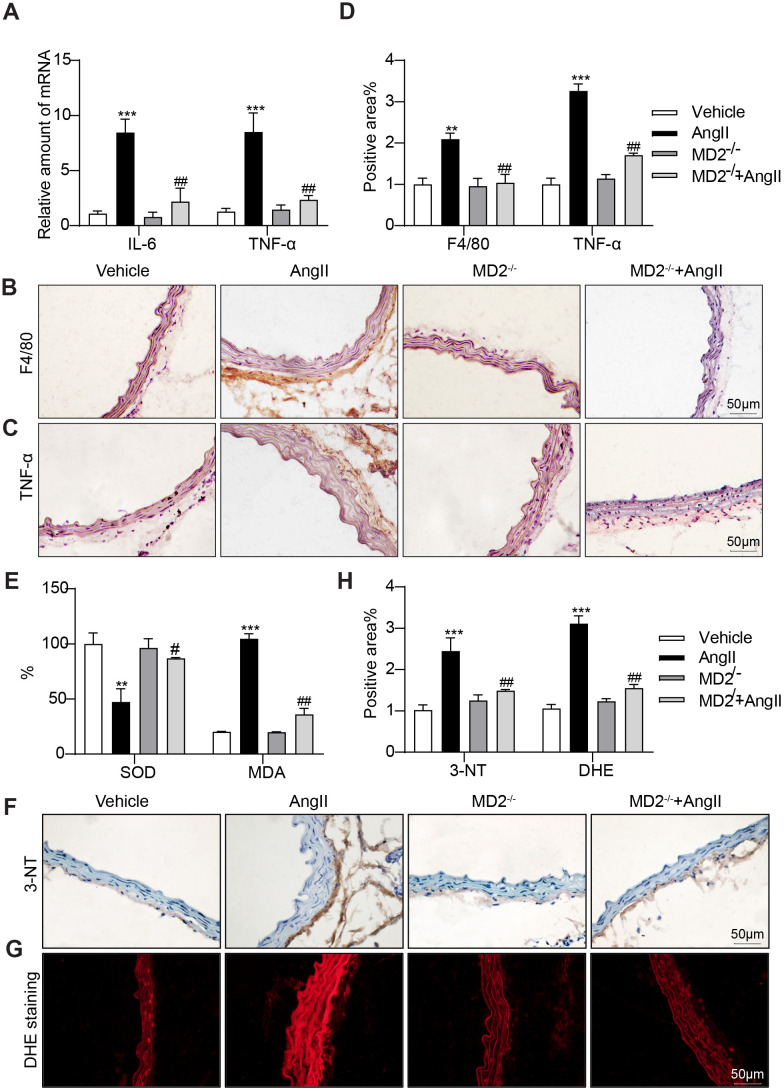
**MD2 deficiency alleviated Ang II induced inflammation and oxidative stress *in vivo*.** (**A**) TNF-α and IL-6 mRNA levels in the aortas were detected using real-time qPCR assay (n = 10; ***p<0.001 compared to Vehicle; ##p<0.01 compared to Ang II). (**B**, **C**) Representative images of F4/80 and TNF-α staining of aortas (scale bar = 50 μm; DAB chromogen staining (brown)). (**D**) Quantification for staining results in 3B-C ((n = 10; **p<0.01, ***p<0.001 compared to Vehicle; ###p<0.001 compared to Ang II). (**E**) Levels of superoxide dismutase (SOD) and malondialdehyde (MDA) in the aortas (n = 10; **p<0.01, ***p<0.001 compared to Vehicle; #p<0.05, ##p<0.01 compared to Ang II). (**F**) Oxidative damage in the aortas as assessed by immunoreactivity to 3-nitrotyrosine (3-NT). Detection was performed by DAB (brown) (scale bar = 50 μm). (**G**) Representative images of dihydroethidium (DHE) staining in the aortas (scale bar = 50 μm). (**H**) Quantification for staining results in 3F-G (n = 10; ***p<0.001 compared to Vehicle; ##p<0.01 compared to Ang II).

As depicted in Figure 3A, the expressing state pertaining to inflammatory cytokines covering interleukin-6 (IL-6) and tumor necrosis factor-α (TNF-α) was significantly increased by Ang II infusion in the WT mice, and these increases were ameliorated by MD2 knockout. Similarly, immunohistochemistry staining against F4/80 and TNF-α showed increased macrophage infiltration and TNF-α expression in the aortas of Ang II-infused WT mice, instead of MD2-KO mice (Figure 3B–3D). This study further determined the levels of inflammatory cell adhesion molecules ICAM-1 and VCAM-1 mRNA in vascular tissues as markers of macrophage infiltration. The results indicated that Ang II treatment increased the ICAM-1 and VCAM-1 mRNA levels in mouse vascular tissues, while MD2 knockout reversed this change (Supplementary Figure 4). In addition, superoxide dismutase (SOD) activity in the aortas was decreased after Ang II-infusion, which was prevented by MD2 knockout (Figure 3E). As a result, oxidative stress markers such as malondialdehyde (MDA), 3-NT and DHE signal showed elevation in WT aortas mice, instead of MD-KO mice after Ang II infusion (Figure 3F–3H).

### MD2 knockdown reduced Ang II-induced collagen deposition and phenotypic switching of VSMCs

**Figure 4:**
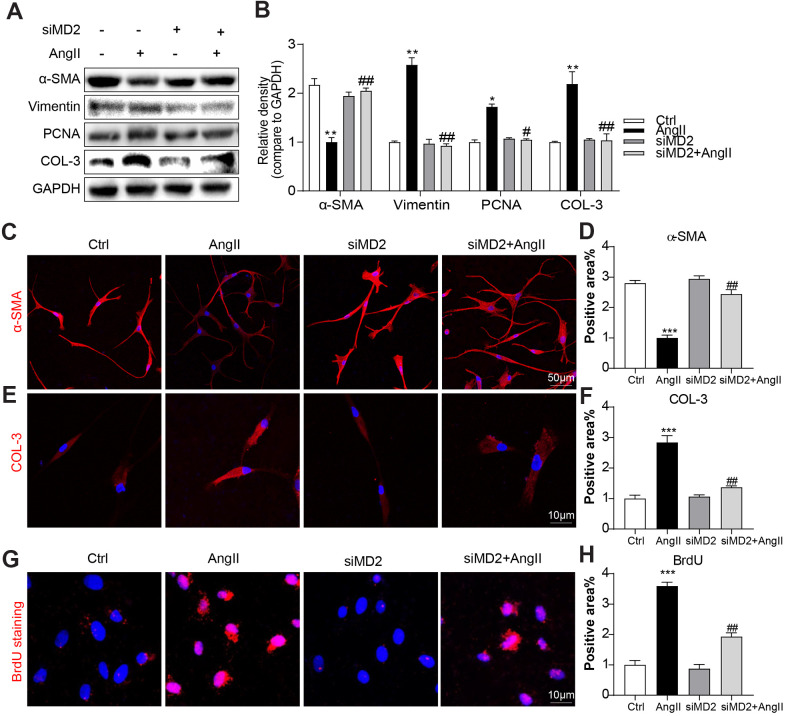
**MD2 knockdown reduced Ang II-induced fibrosis and phenotypic switching in VSMC.** VSMCs were transfected with siRNA against MD2 for 6 h and then incubated with Ang II for 24 h. (**A**, **B**) Expressions of α-SMA, Vimentin, PCNA and COL-3 in the cultural medium were detected by western blot. Densitometric quantification was showed in panel B (n = 3; *p<0.05, **p<0.01 compared to Ctrl; #p<0.05, ##p<0.01 compared to Ang II). (**C**) Representative immunofluorescence staining images for α-SMA (red) in VSMCs. Cells were counterstained with DAPI (blue) (scale bar = 50 μm). (**D**) Quantification for staining results in Figure 4C ((n = 3; ***p<0.001 compared to Ctrl; ##p<0.01 compared to Ang II). (**E**) Representative immunofluorescence staining images for COL-3 (Red) in VSMCs. Cells were counterstained with DAPI (blue) (scale bar = 10 μm). (**F**) Quantification for staining results in Figure 4E (n = 3; ***p<0.001 compared to Ctrl; ##p<0.01 compared to Ang II). (**G**) Proliferation of VSMCs were detected using BrdU staining (red) (scale bar = 10 μm). (**H**) Quantification for staining results in panel G (n = 3; ***p<0.001 compared to Ctrl; ##p<0.01 compared to Ang II).

Consistent with the *in vivo* results, Ang II dose dependently (0.1 to 50 μM) decreased α-SMA protein level in human aortic VSMCs (Supplementary Figure 5A, 5B). Upon Ang II stimulation, VSMCs were changed to a synthetic phenotype, including downregulation of α-SMA expression and upregulation of Vimentin [18]. Human aortic VSMCs underwent the treatment by using Ang II (10 μM) for 24 h under or nor under MD2-targeting siRNA (siMD2) pretreatment for 6 h. MD2 protein level in VSMCs was efficiently knocked down after transfection with siMD2 for 6 h (Supplementary Figure 6A, 6B). As shown in Figure 4A, 4B, pretreatment with siMD2 prevented against Ang II-increased Vimentin, PCNA and COL3 levels and rescued Ang II-decreased α-SMA level in human aortic VSMCs. These effects of siMD2 on α-SMA and COL3 expression were further confirmed by immunofluorescence staining in Ang II-treated VSMCs (Figure 4C–4F). Furthermore, Ang II-induced VSMC proliferation detected by BrdU incorporation was also inhibited by siMD2 (Figure 4G, 4H). These results indicated that MD2 knockdown significantly attenuated collagen deposition, proliferation, and phenotypic switching of VSMCs under the induction of Ang II.

### MD2 knockdown reduced Ang II-induced inflammation and MAPK phosphorylation

**Figure 5:**
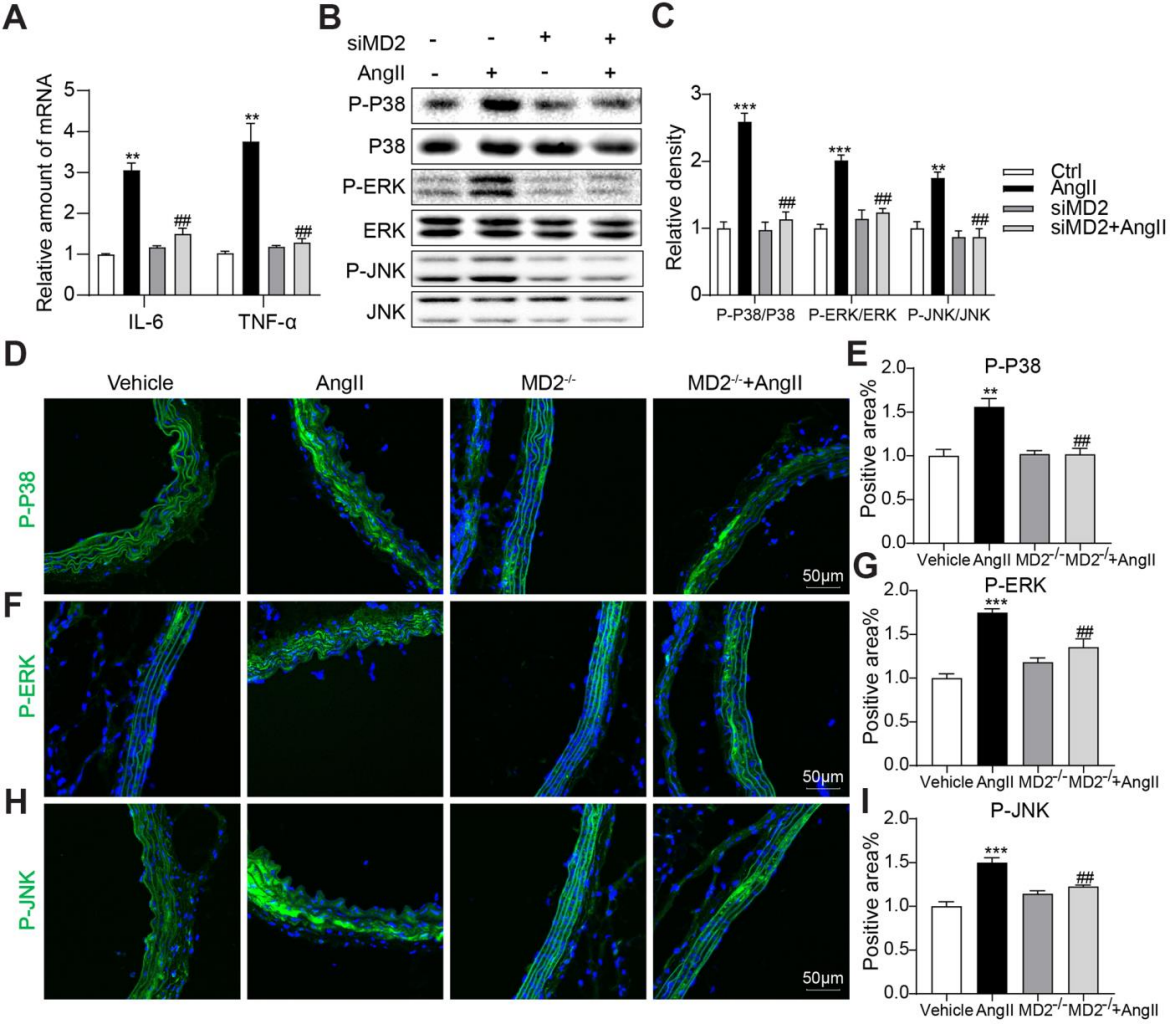
**MD2 knockdown reduced Ang II-induced inflammation by inhibition of MAPKs phosphorylation.** VSMCs were transfected with siRNA against MD2 for 6 h and then incubated with Ang II for 6 h (panel **A**) and 30 min (panel **B**, **C**). (**A**) The level of TNF-α and IL-6 were detected using real-time qPCR assay (n = 3; **p<0.01 compared to Ctrl; ##p<0.01 compared to Ang II). (**B**, **C**) Expressions of P-ERK, P-JNK, P-P38 in the cultural medium were detected by western blot (n = 3; **p<0.01, ***p<0.001 compared to Ctrl; ##p<0.01 compared to Ang II). (**D**–**I**) Representative immunofluorescence staining images and quantification results for P-ERK (green), P-JNK (green), P-P38 (green) in mouse aortas. Tissues were counterstained with DAPI (blue) (scale bar = 50 μm) (n = 10; **p<0.01, ***p<0.001 compared to Vehicle; ###p<0.001 compared to Ang II).

To explore the anti-inflammatory effect of siMD2, human aortic VSMCs received the treatment by using Ang II (10 μM) with or without siMD2 pretreatment. MD2 knockdown significantly inhibited Ang II-induced pro-inflammatory gene expression including IL-6 and TNFα (Figure 5A). Ang II significantly activated MAPKs including P38, ERK and JNK, and MD2 knockdown completely blocked these Ang II-induced MAPK activation (Figure 5B, 5C). These results were confirmed in the aortic tissues from WT and MD2-KO mice administrated with Ang II by immunofluorescent staining of p-JNK, p-ERK, and p-P38. MD2 deficiency prevented Ang II-induced p-JNK, p-ERK, and p-P38 in the aortas *in vivo* (Figure 5D–5I). These results indicated that MD2 defect mitigated inflammation and MAPK phosphorylation under the induction of Ang II.

### MD2 knockdown alleviated Ang II-induced ROS generation and SIRT1 decrease

**Figure 6:**
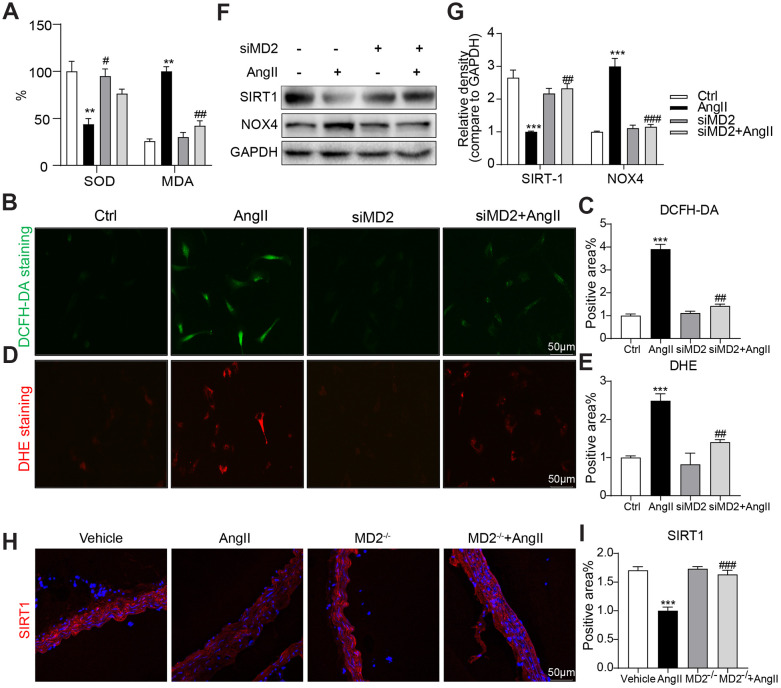
**MD2 knockdown alleviated Ang II-induced ROS by activation of SIRT-1.** (**A**) VSMCs were transfected with siRNA against MD2 for 6 h and then incubated with Ang II for 4 h (panel **A**–**E**) and 24 h (panel **F**–**G**). (**A**) Levels of superoxide dismutase (SOD) and malondialdehyde (MDA) in VSMCs (n = 3; **p<0.01 compared to Ctrl; #p<0.05, ##p<0.01 compared to Ang II). (**C**–**E**) Superoxide production was measured by DCFH-DA (green) and DHE (red) (scale bar = 50 μm) (n = 3; ***p<0.001 compared to Ctrl; ##p<0.01 compared to Ang II). (**F**, **G**) Expressions of SIRT-1and NOX4 in the cultural medium were detected by western blot (n = 3; ***p<0.001 compared to Ctrl; ##p<0.01, ###p<0.001 compared to Ang II). (**H**) Representative immunofluorescence staining images for SIRT-1 (red) in mouse aortas (scale bar = 50 μm). (**I**) Quantification for staining results in panel I (n = 10; ***p<0.001 compared to Vehicle; ###p<0.001 compared to Ang II).

ROS critically impacts Ang II signaling. In human aortic VSMCs, Ang II treating process significantly down-regulated SOD active state while elevating MDA level, and MD2 knockdown prevented Ang II-induced SOD decrease and MDA increase (Figure 6A). These results were parallelly confirmed by directly measuring intracellular ROS levels with DCFH-DA and DHE. The results indicated that Ang II increased DCFH-DA and DHE fluorescence signals in the VSMCs, and MD2 knockdown prevented these increases (Figure 6B–6E). Furthermore, we found that Ang II markedly increased NOX4 but decreased SIRT1 expression, and these changes were blunted by MD2 knockdown in the VSMCs (Figure 6F, 6G). Decreased SIRT1 expression was confirmed in Ang II-infused mouse aortic tissue, and MD2 knockout prevented this decrease (Figure 6H, 6I). As revealed from the mentioned outcomes, MD2 deficiency reduced ROS generation under the induction of Ang II, while increasing SIRT1 expression.

### Pharmacological inhibition of MD2 prevents Ang II-induced injuries in VSMCs

Our further objective was to test whether pharmacological inhibition of MD2 prevents Ang II-induced injuries in VSMCs. We exploited a small-molecule inhibitor of MD2, namely L6H21, binding to MD2 in a direct manner and preventing MD2-mediated TLR4 activation [19]. As expected, pretreatment with L6H21 dose-dependently suppressed protein levels of Vimentin, COL-3, and PCNA, and rescued the expression of α-SMA in Ang II-stimulated VSMCs (Supplementary Figure 7A). The Supplementary Figure 7B showed that the Ang II-increased mRNA levels of TNF-α and IL-6 were also prevented by L6H21 treatment in VMSCs. In the detection of ROS level, L6H21 prevented the increase in DCFH-DA fluorescence signals induced by Ang II in VSMCs (Supplementary Figure 7C). Overall, these data indicated that MD2 blockage by small-molecule inhibitor could reduce oxidative stress, inflammation, and remodeling under the induction of Ang II in VSMCs.

## DISCUSSION

MD2 was found here to robustly increase in the aortic wall of Ang II-induced mouse vascular remodeling model, and the increased MD2 colocalized with α-SMA, one of the makers of VSMCs. MD2 deficiency significantly alleviated vascular remodeling under the induction of Ang II evidenced by reduced collagen depositing process and phenotypic switching pertaining to VSMCs. These protective effects were accompanied with suppressed inflammatory process and oxidation stresses in the aortic wall. These *in vivo* results were further confirmed in Ang II-treated human VSMCs. Furthermore, the protective effects of MD2 deficiency were associated with decreased MAPK phosphorylation and restoration of the suppressed SIRT1 expression in Ang II-induced vascular remodeling.

Previous study has shown that MD2 mediated Ang II- and oxLDL-induced cardiac inflammation and remodeling via directly binding to TLR4 [10, 12]. In addition, MD2 blockage protects obesity-related vascular remodeling via activating AMPK/Nrf2 pathway [20]. The systemin which MD2 critically impacts cardiac inflammation and remodeling under the induction of Ang II was suggested previously [12]. We demonstrated that Ang II directly binds to MD2 and activates TLR4 signaling pathway and inflammation. In this study, we showed that MD2 deficiency alleviated Ang II-induced inflammation and vascular remodeling evidenced by reduced collagen deposition and phenotypic switching of VSMCs. These results indicate that MD2 may be a therapeutic aim in terms of cardiovascular remodeling. We also consider that Ang II directly binds to MD2 and activates inflammatory response in VSMCs, which further leads to vascular fibrosis and remodeling. A pending question arising from our study is how Ang II induces MD2 overexpression in aorta (Figure 1). Since MD2 has been shown to be increased in chronic inflammatory diseases [15, 21, 22], it is possible that inflammation (increased pro-inflammatory cytokines) induces MD2 overexpression in Ang II-challenged mouse aorta in a positive feedback manner. Further, our studies are certainly kept trying to understand the mechanism by which Ang II up-regulate MD2 expression.

Vascular remodeling is a complex process of structural and functional changes of the vascular wall in response to chronic hemodynamic changes, involving a wide variety of cells, e.g. endothelial cells (ECs), SMCs and microphage [23]. Of note, numerous studies have demonstrated the key effect exerted by smooth muscle cell dysfunction within the progress of vascular remodeling [2]. This process is fine-tuned by the RAS. Specifically, Ang II activates NOXs by binding to AT1R and promotes ROS generation, which promotes the transformation of VSMCs to one synthesis-related proliferative type from a quiescent contractile phenotype and eventually leads to the vascular remodeling [24]. Recently, a definite relationship between SIRT1 and ROS was identified [25], and inhibition of SIRT1 up-regulated NOX oxidase subunits, p22^phox^ and NOX4, resulting in increased ROS generation [26]. In this study, MD2 deficiency restored Ang II-induced downregulation of SIRT1 and prevented Ang II-induced NOX4 expression. These may be the underlying mechanisms of decreased oxidative stress in MD2 deficient VSMCs.

In another side, the phenotypic switching of VSMCs is accompanied with increased secretion of inflammation-related cytokines, facilitating the phenotypic switching and ultimately lead to vascular remodeling [27, 28]. Previous studies showed that, in the heart, MD2 knockout reduced the direct binding of Ang II to TLR4 leading to reduced NF-κB nuclear translocation, and ultimately reduced cardiac inflammation and remodeling [12]; in the liver, inhibition of MD2 reduced inflammatory response and attenuated fatty liver disease which is not alcoholic to progress [29]. These studies demonstrate that MD2 may promote inflammation. In this study, we found that MD2 deficiency inhibited inflammatory cytokines expression and MAPKs phosphorylation in Ang II-induced vascular remodeling. Although SIRT1 activation gave the induction of ERK to be phosphorylated inside the keratinocytes and new mouse ventricular cardiomyocytes, according to Becatti et al. [30], SIRT1 activating process is found inhibit JNK and P38 phosphorylating process through AKT deacetylation and ASK1 inhabitation [31]. Considering that MD2 knockout significantly increased SIRT1 expression during Ang II-induced vascular remodeling, the increased SIRT1 level may contribute to the reduction in MAPKs phosphorylation. However, further studies are needed to show the interaction between SIRT1 and MAPKs in the VSMCs. Besides, more studies are needed to explore how MD2 regulates SIRT1 and MAPKs signaling in vascular remodeling.

In summary, MD2 deficiency protects from oxidation stress and inflammation under the induction of Ang II, thereby causing vascular remodeling to be reduced, probably receiving the mediation by elevated SIRT1 and decreased MAPKs phosphorylation levels. MD2 plays a significant role in vascular oxidative stress, inflammation, and remodeling under the induction of Ang II. For this reason, pharmacological inhibition of MD2 acts as a likely target for the treatment of vascular remodeling-mediated cardiovascular diseases.

## MATERIALS AND METHODS

### Cell culture and reagents

Shanghai R&S Biotechnology Co., Ltd. (Shanghai, China) provided the vascular smooth muscle cells (VSMCs). The VSMCs underwent the culture in DMEM covering 100 U/mL of streptomycin, 100 U/mL of penicillin, and 10% fetal bovine serum (FBS) at 37° C in a 5% CO2 incubating element under humidification. Sigma (St. Louis, MO, USA) provided AngII and anti-bromodeoxyuridine (BrdU). Cell Signaling Technology (Danvers, Massachusetts) offered antibodies for P38 (8690S), P-P38 (4511), ERK (9102S), P-ERK (4695S), JNK (9252S), P-JNK (4668S). Abcam (Cambridge, MA) offered MD2 (ab24182), CD31 (ab119341), α-SMA (ab32575), Collagen III (ab-23445), PCNA (ab29), Vimentin (ab8978), 3-NT (ab191308), NOX4 (ab133303), TNF-α (ab-1392), GAPDH (ab-8245), TRITC-conjugated secondary antibody and PE-conjugated secondary antibody. Santa Cruz (CA, USA) offered SIRT1 (sc-74465). The small molecule MD2 inhibitor, L6H21, was synthesized by our lab with a purity of 98.9% [19]. L6H21 was dissolved in dimethylsulphoxide for *in vitro* studies.

### Animal experiments

Male MD2^-^/^-^ mice (B6.129P2-Ly96 knockout [KO], 18-22 g) and wild-type (WT, 18-22 g) littermates with a C57BL/6 background were provided by RIKEN BioResource Center of Japan (Tsukuba, Ibaraki, Japan). Animals were housed with a 12:12 h light–dark cycle at a constant room temperature, and fed a standard rodent diet. The animals were acclimatized to the laboratory for at least 2 weeks before initiating the studies. By complying with the directives outlined in the Guidelines for the Care and Use of Laboratory Animals (US National Institutes of Health), the authors conducted overall animal caring process and experimentally-related process. Animal care and experimental protocols were approved by the Committee on Animal Care of Wenzhou Medical University. The authors employed 20 MD2^-^/^-^ mice and 20 WT mice.

For the development of aortic remodeling system under the induction of Ang II, mice were administered Ang II by subcutaneous injection with micro-pump (1.4 mg/kg/day in phosphate buffer, pH 7.2) for 2 weeks as previously reported [32]. The mentioned mice fell to weight-matched groups in a random manner: (I) no-treated WT control mice that received PBS (control group, n = 10); (II) Ang II-induced vascular remodeling mice (Ang II group, n = 10); (III) no-treated MD2^-^/^-^ control mice that were received PBS (MD2^-^/^-^ group, n = 10); (IV) MD2^-^/^-^ mice that were injection of Ang II (MD2^-^/^-^ + Ang II group, n = 10). Based on the telemetric blood pressure model (BP-2010A, Softron Biotechnology, Tokyo, Japan), blood pressure was measured by tail-cuff [33]. After 2 weeks treatment, animals were sacrificed using sodium pentobarbital anesthesia. The aortas were excised aseptically, blotted dry and the weight was recorded. The tissues were immediate freezing in liquid nitrogen, and then received the storing process at −80° C for subsequent studies.

### Immunohistochemistry

5 μm vascular sections underwent the 30 min treatment using3% H_2_O_2_ and using 1% BSA in PBS for 30 min. Slides received the incubation throughout the night at 4° C with primary antibody (Collagen III, 1:500; PCNA, 1:1000; 3-NT, 1:1000; CD68, 1:1000; TNF-α, 1:500) then incubated with secondary antibody (Santa Cruz; 1:100) for 1 h and DAB (A : B = 1 : 20) for 5 min at ambient temperatures. Lastly, the cell nuclei received the 5 min staining process with hematoxylin, the sections underwent the dehydration, and the images received the viewing process under a light microscope (400× amplification; Nikon, Japan).

### Immunofluorescent staining

5 μm cryostat sections were obtained and put onto gelatin-coated glass slides. Cryostat sections was permeabilized by 0.1% Triton X-100 for 10 minutes and the 30 min blocking process using 2% bovine serum albumin. Next, tissues received the incubation in primary antibodies throughout the night at 4° C and then the fluorescent-labeled secondary antibodies-based 60 min incubating process. Next, the 5 min nuclear staining was conducted using DAPI. Antibody dilutions were prepared as follows: CD31, 1:500; MD2, 1:500; α-SMA, 1:500; Vimentin, 1:500; Collagen III, 1:500; 4’, 6-diamidino-2-phenylindole (DAPI), 1:2000; secondary donkey anti-mouse (488) and donkey anti-rabbit (TRITC) Alexa Fluor–conjugated antibodies, 1:500. This study captured the images based on the Leica A1 laser confocal microscope (Leica, Germany).

### Hematoxylin and eosin (H&E) staining for morphology and Masson’s trichrome staining for fibrosis

Using hematoxylin and eosin (H&E) and Masson, the vascular tissue sections (5 μm) received the staining process for assessing the fibrosis content and intima-media thickness. Next, under the light microscope (400× amplification; Nikon, Japan), the authors conducted the viewing process of the stained sections.

### Determination of superoxide production and the levels of cellular hydrogen peroxide (H_2_O_2_)

Based on dihydroethidium (DHE) staining, superoxide production received the assessment. Briefly, the arterial sections in mice received the excising process, the immediate embedding process in OCT compounds, and the cutting process to 5 μm-thick sections. The Section underwent the incubation in DHE by using PBS (10 mmol/l) under darkness and the humidification-based containing element at 37° C for 45 min. DHE received the oxidization through being reacted to ethidium bromide, binding to DNA in the fluoresces red and nucleus. DHE dilutions were prepared as 1:5000. The images were viewed under the Leica A1 laser confocal microscope (Leica, Germany).

### Determination of malondialdehyde and superoxide dismutase

Based on commercially available tools, the authors conducted the determining process of malondialdehyde (MDA) and superoxide dismutase extents in tissue and cells and by complying with the guidance of the producer (Beyotime Biotech, Nantong, China).

### BrdU immunofluorescence staining

This study performed immunofluorescence staining for measuring cellular proliferation. Specific to cellular proliferating process, VSMCs received the fixing process using 4% paraformaldehyde, the permeabilizing process using 0.1% Triton X-100, and the staining process using BrdU at 30 mg/mL concentration for 1 h. Afterwards, using anti-BrdU antibody (1:50), cells underwent the incubation throughout the night at 4° C. The authors employed TRITC-conjugated secondary antibody (1:200) to conduct the detecting process. Nuclei were stained with the DAPI at ambient temperatures. Under the Leica A1 laser confocal microscope (400× amplification; Leica, Germany), this study conducted the viewing and capturing processes of immunofluorescence.

### Real-time quantitative PCR

Cells received the homogenization in TRIZOL (Thermo Fisher). RNA underwent the extraction by complying with normal protocol. Based on Eppendorf Mastercycler eprealplex detection system (Eppendorf, Hamburg, Germany) and two-step M-MLV Platinum SYBR Green qPCR SuperMix- UDG kit (Thermo Fisher), this study conducted reverse transcribing process and quantitatively-related PCR. Primers were purchased from Thermo Fisher (Shanghai, China) (Supplementary Table 1).

### Western blot assay

Cells were homogenized, and lysed with lysis buffer (AR0101/0103, Boster Biological Technology co.ltd, USA). Using SDS-PAGE gel, lysates received the separation and the electro-transfer to polyvinylidene fluoride membranes. The membranes underwent the blocking process for 1.5 h at ambient temperatures in Tris-buffered saline (TBS), pH 7.6, covering 5 % non-fat milk and 0.05% Tween 20. This study performed primary antibody incubating processes at 4° C throughout the night. Secondary antibodies were applied for 1 h at room temperature. Based on improved enhanced chemiluminescence reagents (Bio-Rad Laboratories, Hercules, CA), immunoreactivity received the visualizing process; then, based on Image J analysis software version 1.38e (NIH, Bethesda, MD, USA), the quantification was conducted. Values received the normalizing process to each protein control.

### Statistical analysis

Overall information denotes 3 individual experimental processes to be means ± SEM. This study carried out the overall statistics-related analyzing processes based on GraphPad Pro. Prism 8.0 (GraphPad, San Diego, CA). The authors conducted t-testing of students and One-way ANOVA accompanied with several comparing testing processes based on Bonferroni correcting process for the analysis of the diversifications of sets of information. P value < 0.05 was of significance.

## Supplementary Material

Supplementary Figures

Supplementary Table 1
