# Supplementary material for: Myeloid differentiation 2 deficiency attenuates AngII-induced arterial vascular oxidative stress, inflammation, and remodeling
Source: Aging (Albany NY). 2021 Jan 20;13(3):4409–27. doi: 10.18632/aging.202402 (PMC7906178; doi:10.18632/aging.202402)
Supplement: Supplementary Figures [file aging-13-202402-s001.pdf]

SUPPLEMENTARY FIGURES

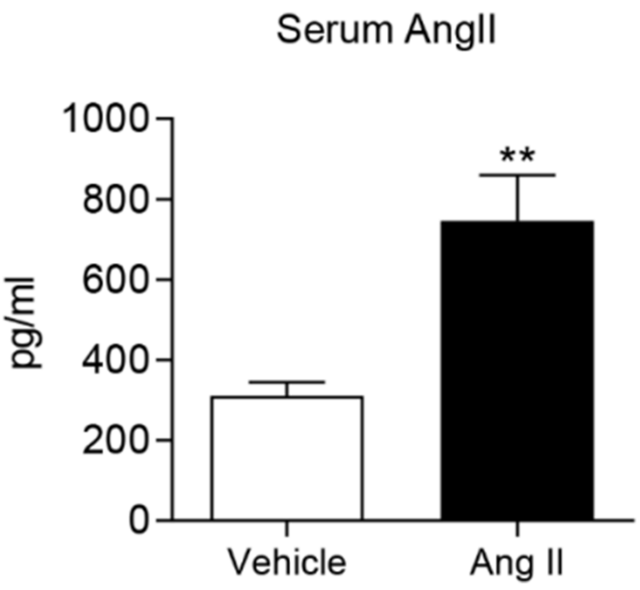

Supplementary Figure 1. AngII levels in serum of AngII-induced mice were dected by ELISA. (n = 10; **P<0.01 compared to Vehicle).

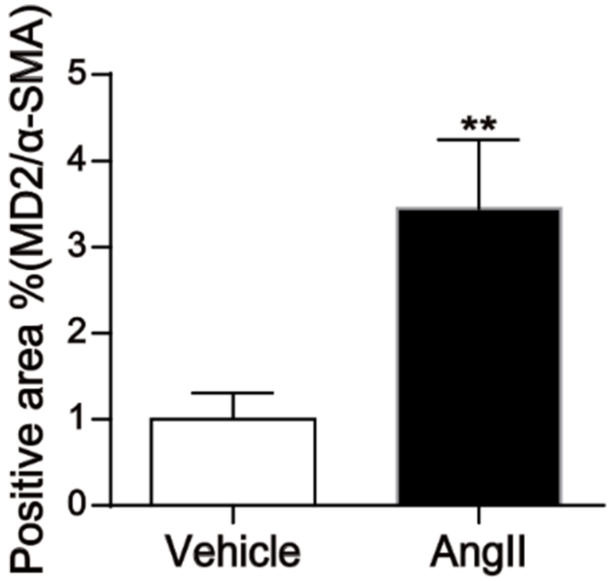

Supplementary Figure 2. Quantification for staining results in Figure 1C (n=8; **P<0.01, compared to Vehicle).

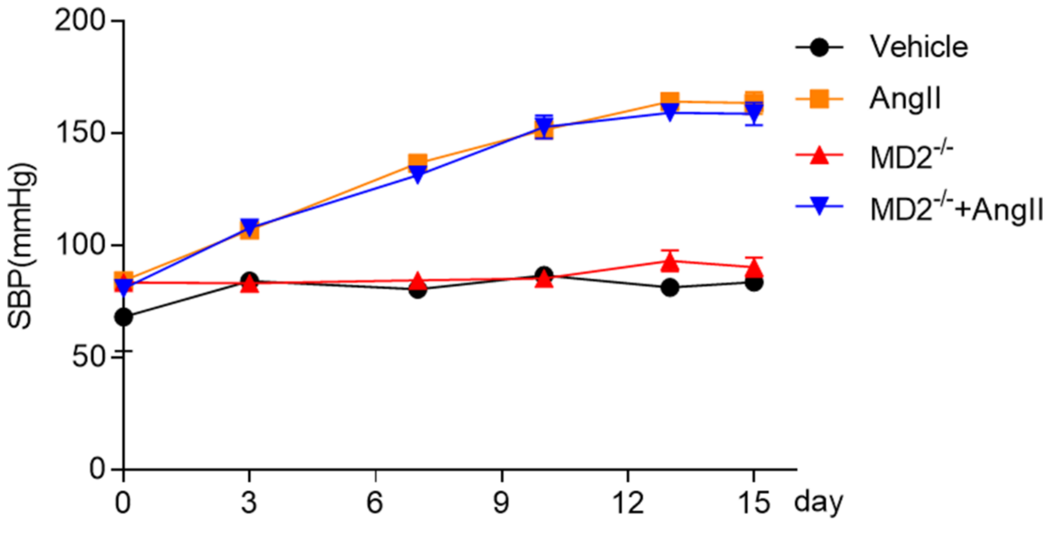

Supplementary Figure 3. Mice blood pressure were measured by tail-cuf using the telemetric blood pressure system every three days.

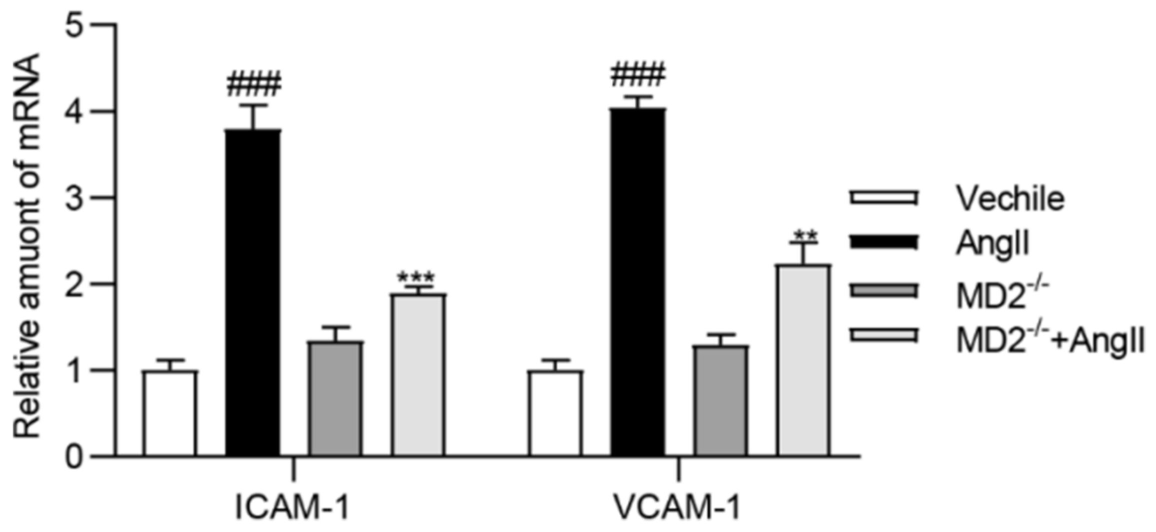

Supplementary Figure 4. MD2 deficiency alleviated Ang II induced ICAM-1 and VCAM-1 transcription in mouse aortas. ICAM-1 and VCAM-1 mRNA levels in the aortas were detected using real-time qPCR assay (n = 10 per group; ###p<0.001 compared to Vehicle; **p<0.01 and ***p<0.0001 compared to Ang II).

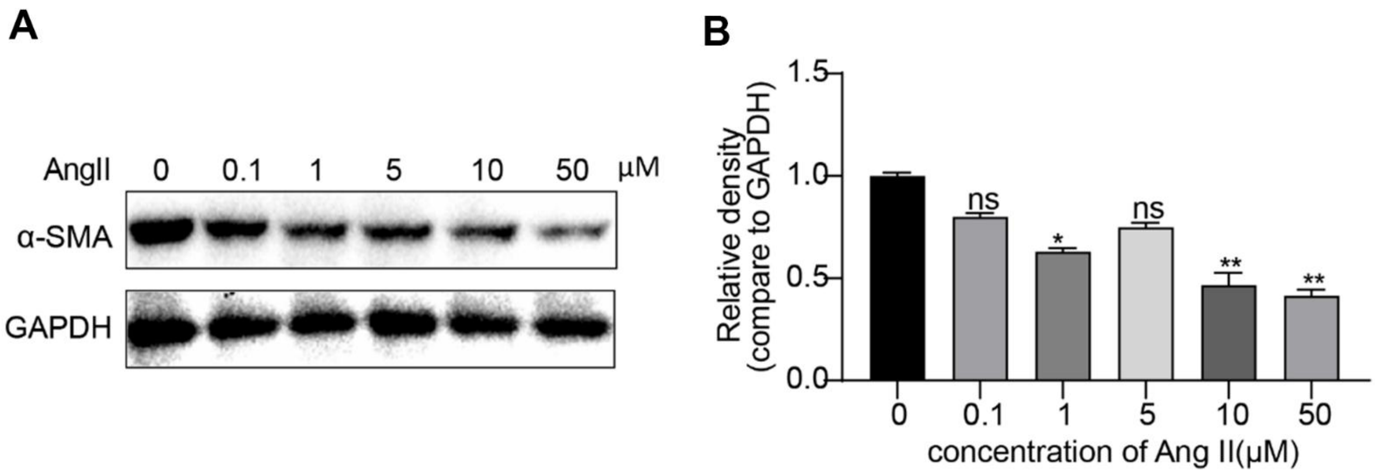

Supplementary Figure 5. (**A**) VSMCs were treated with indicated doses of Ang II for 24 h. The level of α-SMA was detected by western blot. (**B**) Densitometric quantification for panel A (n=3; *P<0.05, **P<0.01, compared to 0).

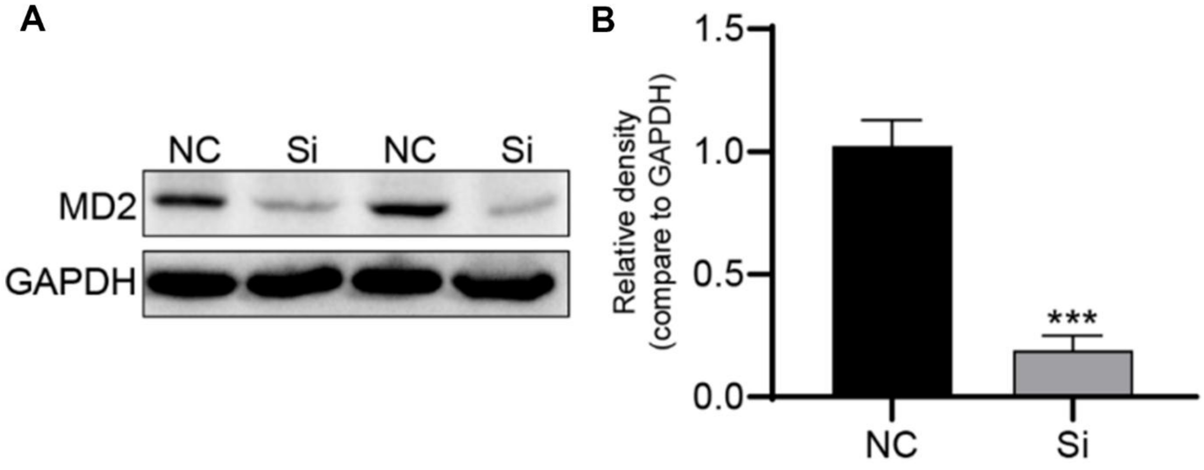

Supplementary Figure 6. (**A**) VSMCs were transfected with siRNA against MD2 for 6 h and then detected expression of MD2 by western blot (two representative data were shown from 3 independent experiments). (**B**) Densitometric quantification for panel A (NC, negative control sequence; Si, siRNA against MD2; n=3; ***P<0.001, compared to NC).

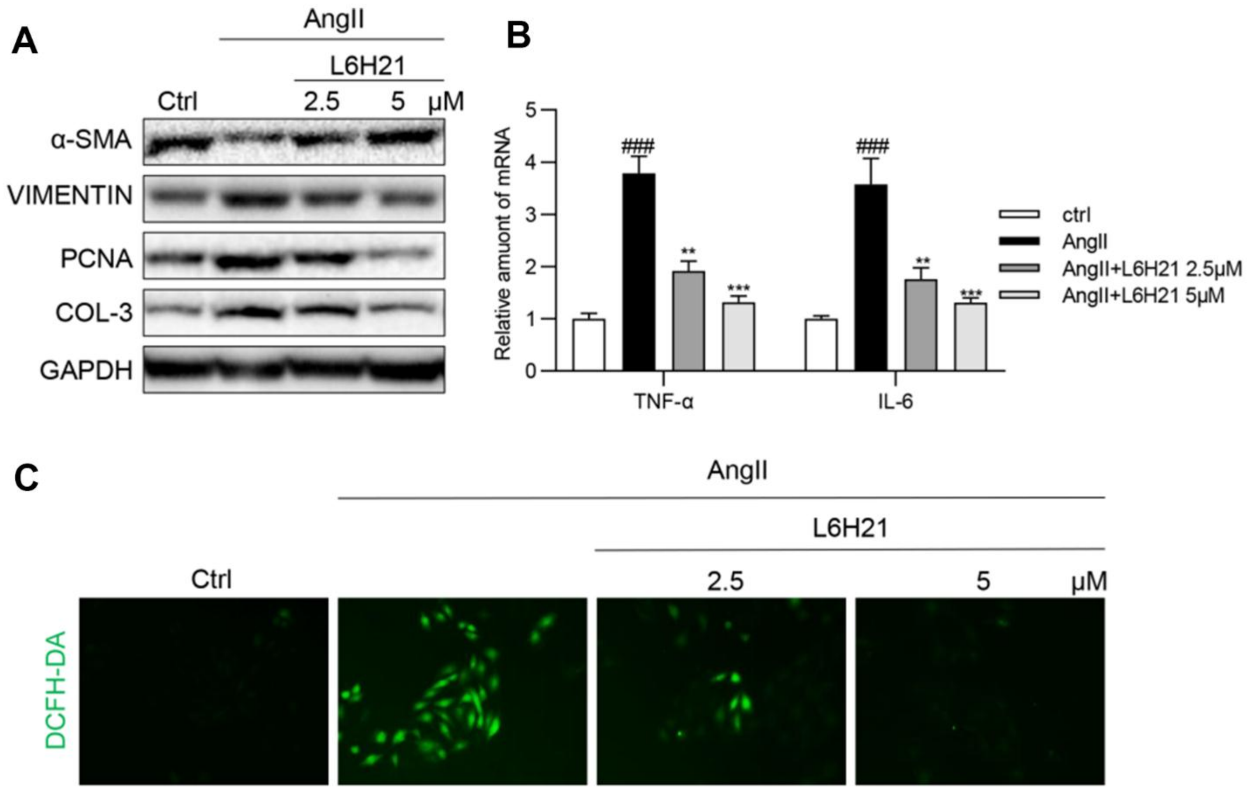

Supplementary Figure 7. MD2 inhibitor L6H21 prevents Ang II-induced injuries in VSMCs. VSMCs were treated with L6H21 (2.5 or 5μM) for 1 hour and then exposed to AngII (10μg/mL) for 24 h (in panels A), 6 h (in panels B), or 12 h (in panels C). (**A**) Expressions of α-SMA, Vimentin, COL-3 and PCNA in cell lysates were detected by western blot analysis. (**B**) The levels of TNF-α and IL-6 mRNA were detected using real-time qPCR assay. (**C**) Superoxide production was measured by DCFH-DA staining (green) (scale bar = 50 μm). Representative blots and images were shown from 3 independent experiments; $^{###}p<0.001$ compared to Ctrl; $^{**}p<0.01$ and $^{***}p<0.001$ compared to Ang II.
