# Supplementary material for: Myeloid differentiation 2 deficiency attenuates AngII-induced arterial vascular oxidative stress, inflammation, and remodeling
Source: Aging (Albany NY). 2021 Jan 20;13(3):4409–27. doi: 10.18632/aging.202402 (PMC7906178; doi:10.18632/aging.202402)
Supplement: Supplementary Table 1 [file aging-13-202402-s002.pdf]

SUPPLEMENTARY TABLE

Supplementary Table 1. Primers used for real-time qPCR assay.

Gene	Species	Primers (FW)	Primers (RW)
TNF-α	Human	CCCAGGGACCTCTCTCTAATC	ATGGGCTACAGGCTTGTCACT
IL-6	Human	ACTCACCTCTTCAGAACGAATTG	CCATCTTTGGAAGGTTCAGGTTG
β-actin	Human	CCTGGCACCCAGCACAAT	GCCGATCCACACGGAGTACT
TNF-α	Mouse	TGATCCGCGACGTGGAA	ACCGCCTGGAGTTCTGGAA
IL-6	Mouse	GAGGATACCACTCCCAACAGACC	AAGTGCATCATCGTTGTTCATACA
β-actin	Mouse	CCGTGAAAAGATGACCCAGA	TACGACCAGAGGCATACAG
